# Hypercaloric Diet Establishes Erectile Dysfunction in Rat: Mechanisms Underlying the Endothelial Damage

**DOI:** 10.3389/fphys.2017.00760

**Published:** 2017-10-04

**Authors:** Iara L. L. de Souza, Bárbara C. Barros, Giuliana A. de Oliveira, Fernando R. Queiroga, Lydiane T. Toscano, Alexandre S. Silva, Patrícia M. Silva, Leylliane F. L. Interaminense, Fabiana de Andrade Cavalcante, Bagnólia A. da Silva

**Affiliations:** ^1^Programa de Pós-graduação em Produtos Naturais e Sintéticos Bioativos, Centro de Ciências da Saúde, Universidade Federal da Paraíba, João Pessoa, Brazil; ^2^Centro de Ciências da Saúde, Universidade Federal da Paraíba, João Pessoa, Brazil; ^3^Departamento de Educação Física, Centro de Ciências da Saúde, Universidade Federal da Paraíba, João Pessoa, Brazil; ^4^Programa de Pós-graduação em Biologia Celular e Molecular, Centro de Ciências Exatas e da Natureza, Universidade Federal da Paraíba, João Pessoa, Brazil; ^5^Departamento de Nutrição, Centro de Ciências da Saúde, Universidade Federal da Paraíba, João Pessoa, Brazil; ^6^Departamento de Fisiologia e Patologia, Centro de Ciências da Saúde, Universidade Federal da Paraíba, João Pessoa, Brazil; ^7^Departamento de Ciências Farmacêuticas, Centro de Ciências da Saúde, Universidade Federal da Paraíba, João Pessoa, Brazil

**Keywords:** obesity, erectile dysfunction, hypercaloric diet, corpus cavernosum, endothelial damage, oxidative stress

## Abstract

Obesity is characterized by an excessive increase in body mass, leading to endothelial damage that may favor the development of erectile dysfunction (ED). ED is defined as the inability to achieve or maintain a penile erection long enough to have a sexual intercourse. In this context, different ED models were developed, however the high price of special animals or the long period to establish the disease has limited studies in this field. Therefore, this study proposed to establish and characterize a novel model of ED in rats associated to a hypercaloric diet consumption. Animals were randomly divided into control group (CG), which received a standard diet, and obese group (OG), fed with a hypercaloric diet during 8 weeks. Rat's erectile function was evaluated *in vivo* and *in vitro*. Food and caloric intake of OG were reduced compared to CG, due to an increased diet energy efficiency. However, OG presented an increased body mass, inguinal, retroperitoneal and epididymal adipose tissues, as well as body adiposity index at the end of experimental protocol. In erectile function analysis, there was a decrease in the number and the latency of penile erections in OG. Additionally, the contractile reactivity of corpus cavernosum was increased in OG, favoring penile detumescence and related to a reduced nitric oxide bioavailability and an increased in contractile prostaglandins levels as a consequence of endothelial damage. Moreover, the endothelium-relaxation reactivity of corpus cavernosum was attenuated in OG associated to the oxidative stress. Thus, it was provided a model for advances in sexual dysfunction field and drug discovery for ED treatment.

## Introduction

Obesity is characterized by an excessive accumulation of adipose tissue located throughout the body and represents a chronic disease that can be caused by multiple factors related to an overeating, a decrease in physical activity, genetic, metabolic, social, behavioral and cultural factors. Currently, it is considered the most common problem of public health in the twenty-first century in both developed and developing countries (Palou et al., 2000; Bowers et al., 2004).

In clinical practice, the classification of overweight and obesity in adults is indicated by the body mass index (BMI), represented by the ratio of weight (kg) and the square of the height (m). Based on these measures, individuals are considered overweight when they present BMI in the range of 25–29.9 kg/m^2^ and obese when BMI is equal to or greater than 30 kg/m^2^ (World Health Organization, 2016).

Obesity triggers changes in endothelial function by chronic elevations in sympathetic activity (Stepp, 2006) and mainly by decreased bioavailability of nitric oxide (NO) due to an increased production of reactive oxygen species (ROS) (Tschöp and Heiman, 2001). In addition, evidence points to the central adiposity as key regulator of vascular endothelial dysfunction (Esposito et al., 2004).

Moreover, obesity alters male fertility and contribute to 45–50% of failures to achieve a successful pregnancy (Lamb and Lipshultz, 2000). Although the cause of this infertility is not fully understood, there is usually a combination of endocrine disorders, defects in the process of spermatogenesis or erectile function (Fullston et al., 2013; Soubry et al., 2013; Mcpherson et al., 2014).

Recent studies have shown that obesity is an independent risk factor for the development of erectile dysfunction (ED) (Esposito et al., 2006, 2008). ED is a multifactorial condition defined as the inability to achieve and/or maintain a penile erection long enough to have a sexual intercourse, and can be found in patients with cardiovascular and endocrine-metabolic diseases, including obesity (Alves et al., 2012). It is associated to an abnormal function in NO signaling pathway, with a consequent reduction of the corpus cavernous relaxation (Lewis et al., 2004).

In the search for new natural or synthetic substances for the treatment of ED, the experimental model of smooth muscle is highlighted, since smooth muscle cells are present in the penis and they are responsible for contraction and relaxation, resulting in flaccidity of penile erection (Webb, 2003).

In addition, in sexual dysfunction field, there are different animal models to study the ED mainly based on nerve stimulation, to measure the intracavernous pressure (ICP); on sexual behavior in conscious animals, such as mating and copulation studies; or on specific predisposing conditions, that usually are aging, radiation, arteriogenic, diabetic or obesity associated ED models (Gajbhiye et al., 2015; Wu and Kovac, 2015).

Thus, experimental models of obesity are presented as a viable tool to investigate organ dysfunctions induced by excessive weight gain, such as erectile dysfunction. Therefore, the purpose of this study was to establish and characterize a novel rat model of erectile dysfunction associated to a hypercaloric diet consumption. Thereby, providing a model for advances in sexual dysfunction field and drug discovery for ED treatment.

## Materials and methods

### Animals

For experimental protocols were used male Wistar rats (*Rattus norvegicus*), 8 weeks of age (approximately 150 g), obtained from the Bioterium Professor Thomas George from Universidade Federal da Paraíba (UFPB). Previously, the animals were maintained in a 12-h light-dark cycle under controlled ventilation and temperature (21 ± 1°C) with free access to water. The experimental procedures were performed following the principles of guidelines for the ethical use of animals in applied etiology studies (Sherwin et al., 2003) and approved by the Ethics Committee on Animal Use of UFPB (protocol n° 0201/14).

### Diets

Animals were randomly divided into two groups (10 animals/group): control (CG) and obese (OG). CG received a standard diet (Presence®) containing by weight 23% protein, 63% carbohydrate and 4% lipids with energy density 3.8 kcal/g. OG received a diet composed by a standard diet (Presence®), milk chocolate, peanuts and sweet biscuit in the proportion of 3:2:2:1. To prepare this diet, all components were powdered and mixed. The diet was prepared weekly and supplied to animals in the form of pellets. The analysis of the hypercaloric diet nutritional composition was performed at the Laboratório de Nutrição Experimental from UFPB. Each experimental group was fed during 8 weeks (Adapted from Estadella et al., 2004).

### Diet centesimal composition and total energy value

The centesimal composition of the diet offered to OG group was determined by analyzes of moisture at 105°C, fixed mineral residue obtained after carbonization and incineration in a muffle furnace at 550°C, proteins by the Kjeldahl method (AOAC, 2002), lipids by the method of Folch, Less and Stanley (Folch et al., 1957), as well as the carbohydrate content (AOAC, 2002). The total energy value (TEV) was calculated using the traditional conversion factors for proteins (4 kcal/g), lipids (9 kcal/g) and carbohydrates (4 kcal/g) (Picchi et al., 2011).

### Chemicals

Magnesium sulfate (MgSO_4_), potassium chloride (KCl), calcium chloride (CaCl_2_), sodium chloride (NaCl) and formaldehyde were purchased from Vetec Química Fina Ltda. (Brazil). Glucose (C_6_H_12_O_6_) and sodium bicarbonate (NaHCO_3_) were purchased from Dinâmica (Brazil). Potassium monobasic phosphate (KH_2_PO_4_), sodium hydroxide (NaOH) and hydrochloric acid (HCl) were purchased from Nuclear (Brazil). These substances, except glucose, NaCl and NaHCO_3_ were diluted in distilled water to obtain each solution, which were maintained under refrigeration.

Phenylephrine (Phe) was purchased from Pfizer (USA). Acetylcholine (ACh), sodium nitroprusside (SNP), L-N^ω^-nitroarginine methyl ester (L-NAME), indomethacin, tempol, apocynin, Cremophor® and R-(-)-apomorphine were obtained from Sigma-Aldrich (Brazil). All substances were diluted in distilled water as needed for each experimental protocol. The carbogen mixture (95% O_2_ and 5% CO_2_) was acquired from White Martins (Brazil).

### Evaluation of food intake and animal's weight gain

The food intake was calculated daily and represents the difference between the offered and residual food: intake (g) = offered quota (g) – clean reject (g). The food that was not eaten and remained in the outer area of the cage was considered a clean reject (Vadivel and Pugalenthi, 2010). The body mass (g) of the animals was recorded 2 times per week, and the weight gain was calculated by the difference between the final and initial body mass.

### Evaluation of diet's energy efficiency

Energy efficiency ratio of the diet was calculated as the rate of body weight gain (g) and the total energy consumption (kcal) of animals after the 8-week period (Feinman and Fine, 2007).

### Experimental assessment of the obesity induction

#### Murinometrics parameters

At the end of 8-weeks, animals were weighed and the naso-anal length was measured to calculate the Lee index as the cubic root of the body mass divided by the naso-anal length and the BMI as the ratio between body weight (g) and the square of body length (cm^2^) (Lee, 1929; Novelli et al., 2007).

#### Mass of white adipose tissue

Twenty-four hours after the last exposure to diet, rats were euthanized by guillotine and the adipose inguinal, retroperitoneal and epididymal tissues were careful dissection and weighed. These white adipose tissues (WAT) represent the main constituents of the central adiposity (Mauer et al., 2001).

#### Body adiposity index

Body adiposity index was calculated by the sum of adipose inguinal, retroperitoneal and epididymal tissues weight, using the formula: inguinal + retroperitoneal + epididymal fat × 100/final body weight (Stunkard and Wadden, 1993).

#### Adipose tissue morphometry

Samples of WAT were fixed in 10% formaldehyde solution, subjected to standard histological procedures as follow: (1) dehydration, by increasing alcohol series of 70% for 24 h, 80, 96, and 100% (third bath) for 1 h each; (2) diaphanization, by bath in 100% xylene alcohol (1:1) for 1 h, followed by two baths in pure xylene for 1 h each; and (3) embedding in paraffin by passing the WAT through two baths of liquid paraffin (heated to 50°C) for 1 h each. Then, WAT were embedded in a new paraffin. The blocks obtained were cut to 5 μm thick and stained with Mayer's hematoxylin/eosin (Howard et al., 2004). Digital images of histological sections were obtained, and about 100 adipocytes per animal had its diameter measured using the Leica Qwin 3.1 software.

### Biochemical analysis

After 12 h fasting, the rat blood was collected from the end tail section. Blood glucose measurement by glucometer was performed using reactive test strips in the first and last day of the dietary protocol (Nayeri et al., 2014). For total cholesterol and triglycerides measurement, the rat blood was collected by cardiac puncture. Therefore, blood was placed in test tubes containing EDTA to obtain the plasma (Okafor et al., 2011; Silva et al., 2012). Then, samples were centrifuged at 0.02 G for 15 min and the supernatant was transferred to Eppendorfs® at –80°C. Analysis were performed using specific commercial kits Labtest® (Minas Gerais, Brazil), according to manufacturer standards, on the automatic biochemical analyzer LabMax 240 (Minas Gerais, Brazil).

### Penile erection induction

For acclimatization, animals were individually placed in a box during 30 min, then received a dorsal subcutaneous injection of apomorphine (80 mg/kg) prepared in saline solution (NaCl 0.9%) or saline solution as vehicle. Subsequently, animals were filmed for 30 min and through the images were evaluated the latency time for achieve the first erection and the number of erections obtained for each animal. Erections were characterized by events where it was possible to observe penile erection, accompanied by lordosis, in which the animal rests on its hind legs, leaning the body forward, holding the penis and licking the organ. Other observed behaviors were counted as licks (Matsumoto et al., 2005).

### Corpus cavernosum isolation

Animals were euthanized as described at section Mass of White Adipose Tissue. Corpus cavernosum was immediately removed, cleaned of fat and connective tissue, immersed in physiological solution at room temperature and bubbled with carbogen mixture. To register isometric contractions, corpus cavernosum segments (1 cm) were suspended in steel rods in organ baths (6 mL), connected to a force transducer (TIM 05), attached to an amplifier (AECAD04F) and connected to an A/D converter into a PC running AQCAD® software (São Paulo, Brazil). The system contained a thermostatic pump model BT 60 that controlled the organ baths temperature.

The physiological solution used was Krebs solution, whose pH was adjusted to 7.4, and the composition (in mM) was: NaCl (118.4), KCl (4.7), CaCl_2_ (2.5), MgSO_4_ (1.2), NaHCO_3_ (25.0), KH_2_PO_4_ (1.17), D-glucose (5.6). Corpus cavernosum was stabilized for 1 h under a resting tension of 0.5 g at 37°C and bubbled with a carbogen mixture (Claudino et al., 2004).

### Contractile reactivity measurement

Corpus cavernosum was assembled as described in section Corpus Cavernosum Isolation. After the stabilization period, cumulative concentration-response curves were obtained to Phe (10^−8^ × 10^−3^ M) in absence or either in the presence of L-NAME 10^−4^ M, an inhibitor of nitric oxide synthase (NOS) (Vignozzi et al., 2006) or indomethacin 10^−5^ M, a cyclo-oxigenase inhibitor (Cartledge et al., 2000), during 30 min of incubation. The contractile reactivity was evaluated based on the values of the negative logarithm of the molar concentration of a substance that produced 50% of its maximal effect (pD_2_) and maximum effect (E_max_) of contractile agent, calculated from the concentration-response curves obtained. The maximum amplitude obtained from the control concentration-response curve was elected as 100% of contraction and the others percentages of contraction were calculated related to this value.

### Relaxing reactivity measurement

Corpus cavernosum was assembled as described in section Corpus Cavernosum Isolation. After the stabilization period, a contraction was induced with Phe 10^−5^ M and performed a cumulative concentration-response curves to ACh (10^−12^–10^−3^ M) or SNP (10^−11^–10^−4^ M), in different preparations. The curves to ACh were obtained in absence or either in the presence of tempol 10^−3^ M, a superoxide dismutase mimetic (Peixoto et al., 2009) or apocynin 10^−4^ M, an inhibitor of NADPH oxidase (Côco et al., 2016), during 30 min of incubation. The relaxing reactivity was evaluated based on the values of pD_2_ and E_max_, calculated from the concentration-response curves obtained.

### Assessment of lipidic peroxidation levels

To identify possible changes in the lipid matrix and cell membranes resulting from the indirect effect of ROS production, was used a technique that detect the reaction of thiobarbituric acid (TBARS) with the products of decomposition of hydroperoxide (Ohkawa et al., 1979). Lipid peroxidation was determined measuring the chromogenic product of the 2-thiobarbituric acid (TBA) reaction with malondialdehyde (MDA) (Winterbourn et al., 1985). Plasma samples (250 μL) was precipitated with 400 μL of 35% perchloric acid and centrifuged at 0.02 G for 20 min at 4°C. The supernatant was collected and 400 μL of 0.6% TBA was added and incubated at 95–100°C for 1 h. After cooling, the samples were read in a spectrophotometer at a wave length of 532 nm (Biospectro, SP-220 model-Brazil). The determination of the MDA concentration was made by substituting the absorbance values in the MDA standard curve obtained on the basis of a standard solution (1 μL of 1,1,3,3-tetramethoxypropane in 70 mL distilled water) diluted in series of 250; 500; 750; 1,000; 1,250; 1,500; 1,750; 2,000; 2,250; 2,500; 2,750, and 3,000 μL of distilled water.

### Evaluation of antioxidant activity

The procedure was based on the method described by Brand-Williams et al. (1995). Briefly, an aliquot of 1.25 mg of DPPH was diluted in 100 mL of ethanol, kept under refrigeration and protected from light. In appropriate centrifuge tubes were added 3.9 mL of DPPH solution and 100 μL of plasma. The tubes were vortexed and left to stand for 30 min. Then, were centrifuged at 0.02 G at 20°C for 15 min and the supernatant was used in a spectrophotometer at 515 nm (Biospectro, model SP-220/Brazil). Results were expressed as percentage of the inhibition of the oxidation: AOA = 100 − ((DPPH • R)_S_/(DPPH • R)_W_ 100). Where (DPPH • R)_S_ and (DPPH • R)_W_ corresponding to the concentration of DPPH • remaining after 30 min, measured in the sample (S) and white (W) prepared with distilled water.

### Statistical analysis

Results were expressed as the mean and standard error of the mean (S.E.M.) and statistically analyzed using Student's *t*-test to intergroup comparison or one-way analysis of variance (ANOVA) followed by Tukey's post-test to intragroup comparison. In order to verified the correlation between the variables was used the Pearson's correlation coefficient (r). Values were significantly different when *p* < 0.05. All data were analyzed by GraphPad Prism® version 5.01 (GraphPad Software Inc., San Diego, CA, U.S.A.).

## Results

### Diet centesimal composition and total energy value

The centesimal composition of the experimental diet analyzed in this study as well as its TEV were showed in Table 1.

**Table 1:** Centesimal composition of the experimental diets.

**Parameters**	**Mean ± S.E.M**.
Moisture (%)	10.5 ± 0.003
Ashes (%)	4.6 ± 0.05
Carbohydrate (%)	45.5 ± 0.02
Protein (%)	22.8 ± 0.01
Lipid (%)	16.0 ± 0.02
TEV (kcal/g)	4.2 ± 0.001

### Evaluation of food intake and animal's weight gain

The weekly food intake of the CG and OG groups did not differ in the 1st week (145.0 ± 7.8 vs. 125.5 ± 5.9 g, respectively). However, from the 2nd week onwards, there was a decrease in food intake of OG (136.1 ± 3.4; 136.7 ± 5.0; 132.4 ± 5.0; 115.4 ± 2.2; 106.3 ± 4.9; 116.4 ± 4.2 and 120.4 ± 5.9 g, respectively) when compared to CG (164.3 ± 8.0; 167.2 ± 5.8; 169.5 ± 7.5; 168.0 ± 5.2; 167.6 ± 7.1; 185.3 ± 6.5 and 177.0 ± 8.0 g, respectively) (Table 2, *n* = 10). Meanwhile, the weekly caloric intake of the CG and OG groups was similar in the 1st (551.2 ± 29.7 vs. 125.5 ± 5.9, respectively) and 2nd weeks (624.20 ± 30.5 vs. 567.3 ± 14.0, respectively). In addition, between the 3rd and the 8th weeks, there was a decrease in the caloric intake of the OG (570.1 ± 21.0; 552.0 ± 21.0; 479.2 ± 10.0; 443.2 ± 20.5; 485.2 ± 17.6 and 502.2 ± 10.5 kcal, respectively) when compared to CG (635.3 ± 22.0; 644.2 ± 28.4; 638.2 ± 19.8; 636.9 ± 26.9; 704.0 + 24.7 and 672.4 ± 30.5 kcal, respectively) (Table 3, *n* = 10, one-way ANOVA followed by Tukey's post-test).

**Table 2:** Estimated weekly food intake (g) for both CG and OG groups.

**Time (week)**	**Estimated food intake (g)**	
	**CG**	**OG**
1	145.0 ± 7.8	125.5 ± 5.9
2	164.3 ± 8.0	136.1 ± 3.4^*^
3	167.2 ± 5.8	136.7 ± 5.0^*^
4	169.5 ± 7.5	132.4 ± 5.0^*^
5	168.0 ± 5.2	115.4 ± 2.2^*^
6	167.6 ± 7.1	106.3 ± 4.9^*^
7	185.3 ± 6.5^#^	116.4 ± 4.2^*^
8	177.0 ± 8.0	120.4 ± 5.9^*^

*One-way ANOVA followed by Tukey's post-test*,

# *p < 0.05 (CG 1st week vs. CG 7th week). Student's t-test*,

* *p < 0.05 (CG 2nd week vs. OG 2nd week, CG 3rd week vs. OG 3rd week, CG 4th week vs. OG 4th week, CG 5th week vs. OG 5th week, CG 6th week vs. OG 6th week, CG 7th week vs. OG 7th week and CG 8th week vs. OG 8th week) (n = 10)*.

**Table 3:** Caloric weekly food intake (kcal) for both CG and OG groups.

**Time (week)**	**Caloric food intake (kcal)**	
	**CG**	**OG**
1	551.2 ± 29.7	522.2 ± 24.7
2	624.2 ± 30.5	567.3 ± 14.0
3	635.3 ± 22.0	570.1 ± 21.0^*^
4	644.2 ± 28.4	552.0 ± 21.0^*^
5	638.2 ± 19.8	479.2 ± 10.0^*^
6	636.9 ± 26.9	443.2 ± 20.5^*^
7	704.0 ± 24.7^#^	485.2 ± 17.6^*^
8	672.4 ± 30.5	502.2 ± 10.5^*^

*One-way ANOVA followed by Tukey's post-test*,

# *p < 0.05 (CG 1st week vs. CG 7th week). Student's t-test*,

* *p < 0.05 (CG 3rd week vs. OG 3rd week, CG 4th week vs. OG 4th week, CG 5th week vs. OG 5th week, CG 6th week vs. OG 6th week, CG 7th week vs. OG 7th week and CG 8th week vs. OG 8th week) (n = 10)*.

CG showed a total food intake of 1342.0 ± 39.9 g in the 8-week period, corresponding to a total caloric intake of 5098.0 ± 151.9 kcal, and a mean weekly food intake of 168.0 ± 2.6 g, resulting in an average weekly caloric intake of 638.3 ± 10.1 kcal (Table 4, *n* = 10). Nonetheless, OG presented a total food intake of 952.8 ± 13.7 g at the end of the experimental period, resulting in a total caloric intake of 3973.0 ± 57.1 kcal, and a mean weekly food intake of 123.6 ± 1.9 g, corresponding to an average weekly caloric intake of 515.2 ± 7.8 kcal (Table 4, *n* = 10, one-way ANOVA followed by Tukey's post-test).

**Table 4:** Mean and total values of food intake (g) and caloric intake (kcal) for both CG and OG.

**Group**	**Average food intake (g)**	**Total food intake (g)**	**Average caloric intake (g)**	**Total caloric intake (g)**
CG	168.0 ± 2.6	1342.0 ± 39.9	638.3 ± 10.1	5098.0 ± 151.9
OG	123.6 ± 1.9^*^	952.8 ± 13.7^*^	515.2 ± 7.8^*^	3973.0 ± 57.1^*^

*Student's t-test*,

* *p < 0.05 (CG vs. OG) (n = 10)*.

**Figure 1:**
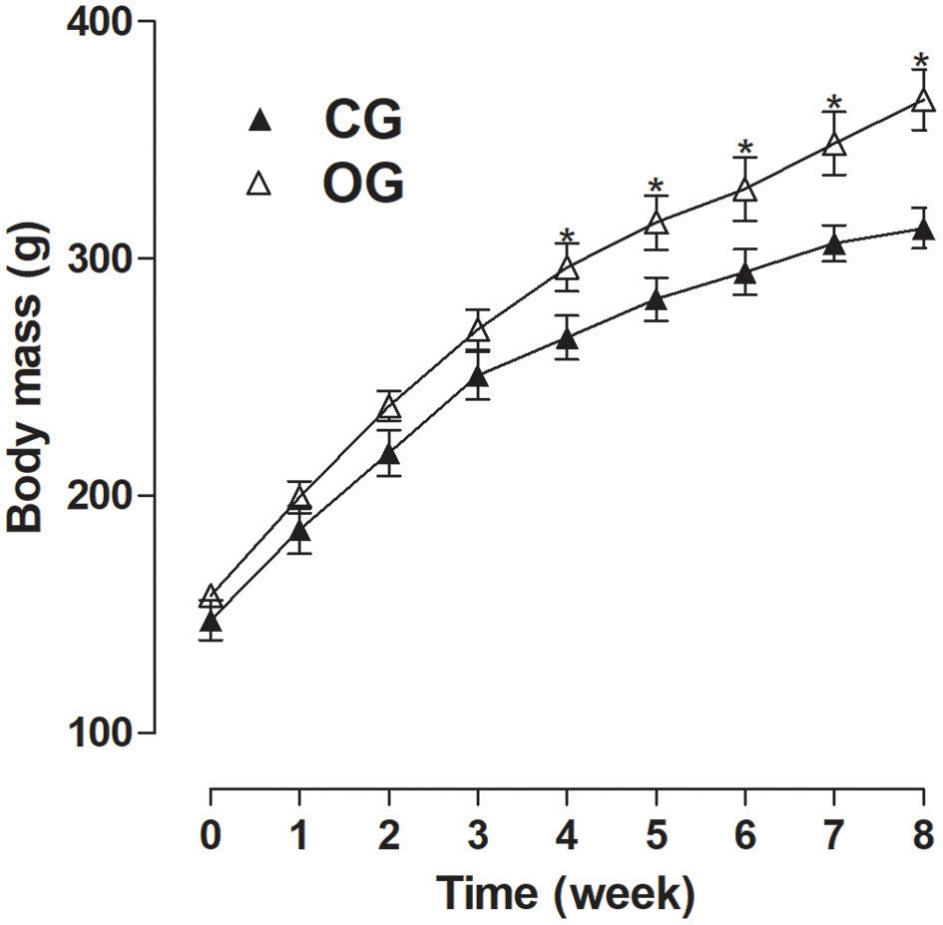
Body mass (g) for both CG (▴) and OG (▵). The symbols and vertical bars represent the mean and S.E.M., respectively (*n* = 10). Student's *t*-test, ^*^p < 0.05 (CG 4th week vs. OG 4th week, CG 5th week vs. OG 5th week, CG 6th week vs. OG 6th week, CG 7th week vs. OG 7th week, CG 8th week vs. OG 8th week).

CG presented an initial body mass of 147.6 ± 8.4 g, not differing from OG, which showed an initial body mass of 158.2 ± 2.4 g. However, after the 8-week experimental period, there was an increase in OG's final body mass in relation to CG (367.1 ± 12.8 vs. 311.9 ± 8.2 g, respectively). In addition, the OG presented a higher body mass from the 4th week of consumption of the experimental diet (296.4 ± 10.1, 315.3 ± 11.5, 329, 4 ± 13.3, 348.7 ± 13.3 and 367.1 ± 12.8 g, respectively) compared to CG (266.8 ± 9.2, 283.0 ± 9.1, 294.4 ± 9.6, 306.6 ± 7.7, and 312.9 ± 8.5 g, respectively) (Figure 1, *n* = 10, one-way ANOVA followed by Tukey's post-test).

### Evaluation of diet's energy efficiency

The energy efficiency ratio of the standard diet offered to CG (3.0 ± 0.3 g/kcal × 100) was lower than the diet offered to OG (5.2 ± 0.3 g/kcal × 100) (*n* = 10, Student's *t*-test).

### Experimental assessment of the obesity induction

#### Murinometrics parameters

CG presented a naso-anal length of 23.2 ± 0.3 cm, differing from the OG (24.6 ± 0.3 cm), however, the Lee index of both CG and OG was similar (0.3 ± 0.004 and 0.3 ± 0.002 g/cm, respectively) as well as the BMI (0.58 ± 0.04 and 0.60 ± 0.03 g/cm^2^, respectively) (*n* = 10, Student's *t*-test).

#### Mass of white adipose tissue

**Figure 2:**
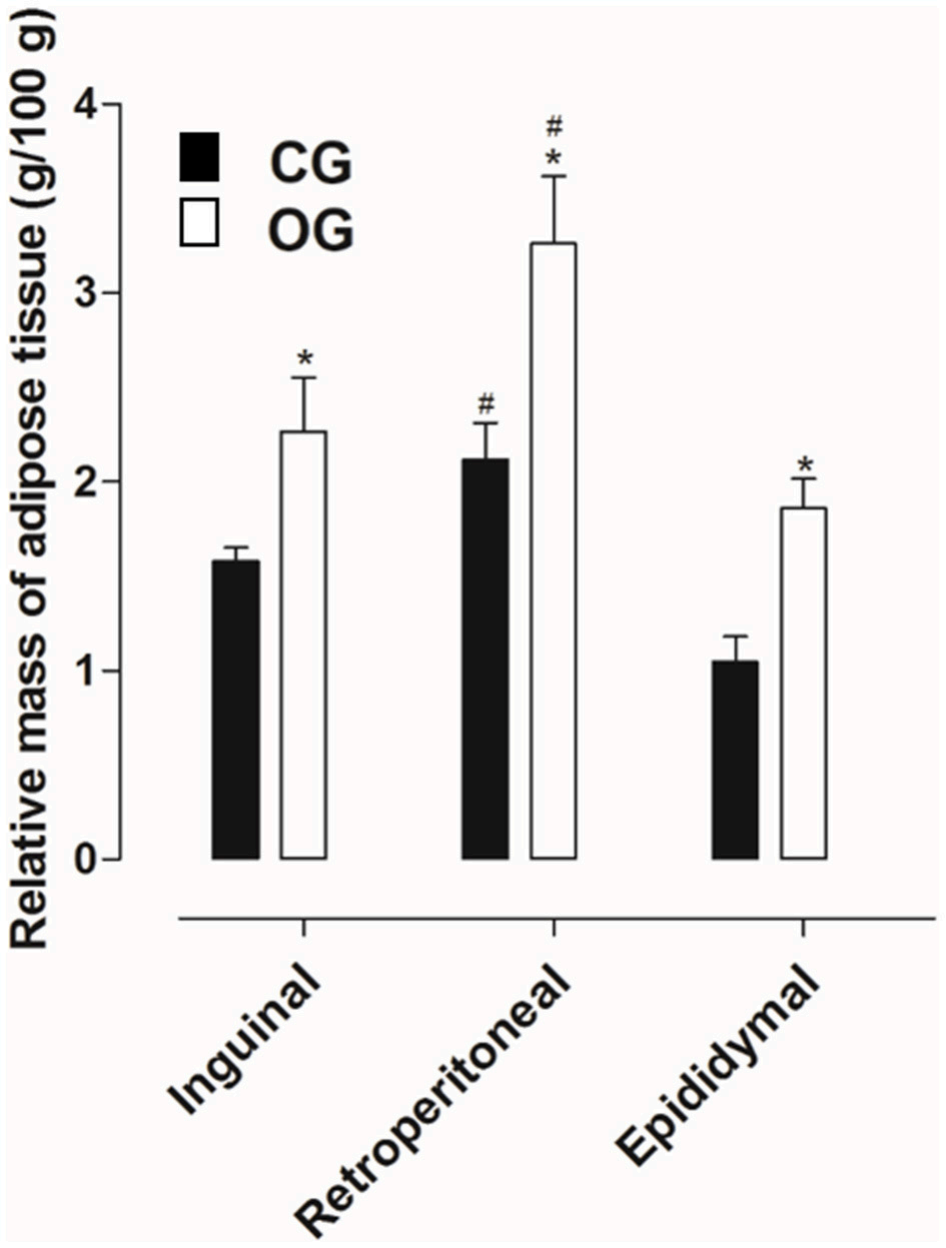
Relative mass of the inguinal, retroperitoneal and epididymal adipose tissues (g/100 g) of rats from both CG (■) and OG (□). The columns and vertical bars represent the mean and S.E.M., respectively (*n* = 10). One-way ANOVA followed by Tukey's post-test, ^#^*p* < 0.05 (CG retroperitoneal vs. CG epididymal, OG inguinal vs. OG retroperitoneal and OG retroperitoneal vs. OG epididymal). Student's *t*-test, ^*^p < 0.05 (CG inguinal vs. OG inguinal, CG retroperitoneal vs. OG retroperitoneal and CG epididymal vs. OG epididymal).

Inguinal, retroperitoneal and epididymal adipose tissues presented higher mass in OG (2.3 ± 0.3, 3.3 ± 0.3, and 1.8 ± 0.1 g/100 g, respectively), compared to CG (1.6 ± 0.1, 2.1 ± 0.2, and 1.0 ± 0.1 g/100 g, respectively) (Figure 2, *n* = 10, one-way ANOVA followed by Tukey's post-test).

#### Body adiposity index

CG showed a body adiposity index lower than the OG (1.5 ± 0.1 vs. 2.0 ± 0.1) (*n* = 10, Student's *t*-test).

#### Adipose tissue morphometry

**Figure 3:**
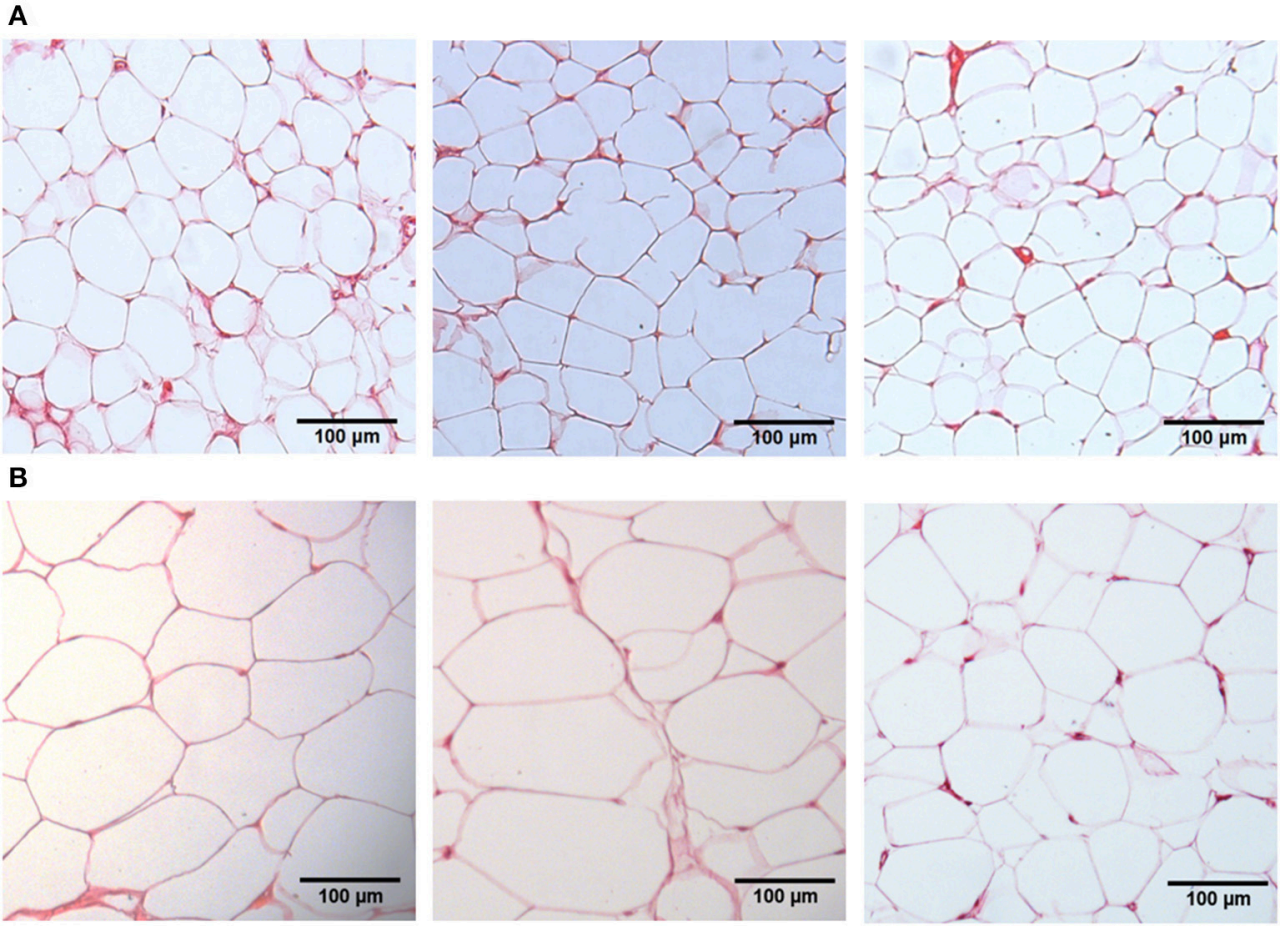
Microphotography of inguinal, retroperitoneal and epididymal adipocytes (μm) of rats from both CG **(A)** and OG **(B)** groups. Increased lens 20×.

**Figure 4:**
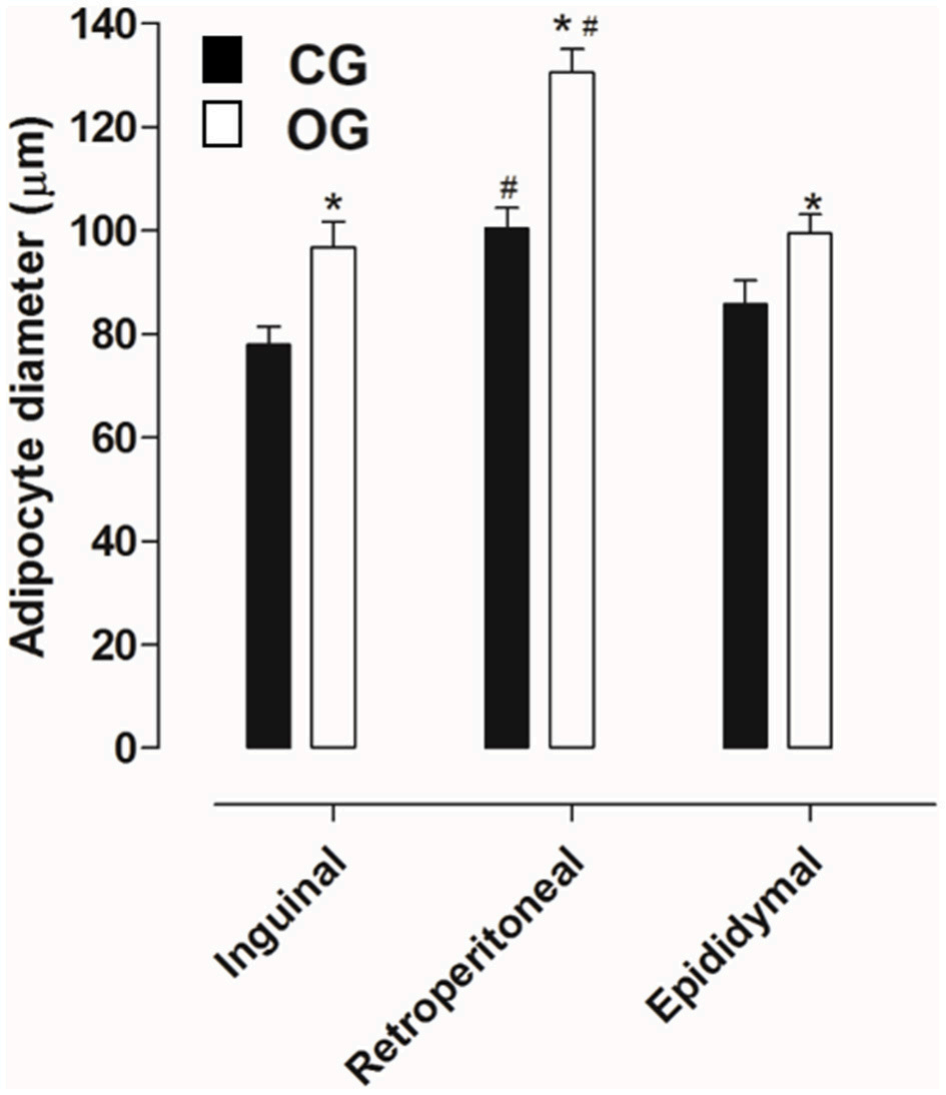
Diameter of the inguinal, retroperitoneal and epididymal adipocyte (g/100 g) of rats from both CG (■) and OG (□). The columns and vertical bars represent the mean and S.E.M., respectively (*n* = 5). One-way ANOVA followed by Tukey's post-test, ^#^*p* < 0.05 (CG inguinal vs. CG retroperitoneal, CG epididymal vs. CG retroperitoneal, OG inguinal vs. OG retroperitoneal and OG epididymal vs. OG retroperitoneal). Student's *t*-test, ^*^p < 0.05 (CG inguinal vs. OG inguinal, CG epididymal vs. OG epididymal and CG retroperitoneal vs. OG retroperitoneal).

Inguinal, retroperitoneal and epididymal adipocytes obtained from CG (77.8 ± 3.6, 100.4 ± 3.9, and 85.7 ± 4.7 μm, respectively) presented diameters lower than that registered in the OG (96.7 ± 5.0, 130.5 ± 4.5, and 99.4 ± 3.6 μm, respectively). The largest diameter was observed in retroperitoneal adipocytes in both groups (Figures 3, 4, *n* = 5, one-way ANOVA followed by Tukey's post-test).

### Biochemical analysis

CG showed an initial and final fasting blood glucose of 88.1 ± 2.3 and 86.9 ± 4.5 mg/dL, respectively. Similarly, an initial and final fasting blood glucose of 86.2 ± 2.6 and 91.4 ± 3.1 mg/dL, respectively, were observed in OG (*n* = 10). In addition, total cholesterol (52.2 ± 3.4 vs. 53.0 ± 3.1 mg/dL, respectively) and triglycerides levels (72.4 ± 6.1 vs. 83.3 ± 6.6 mg/dL, respectively) were not different in CG and OG (*n* = 10, Student's *t*-test).

### Penile erection induction

**Figure 5:**
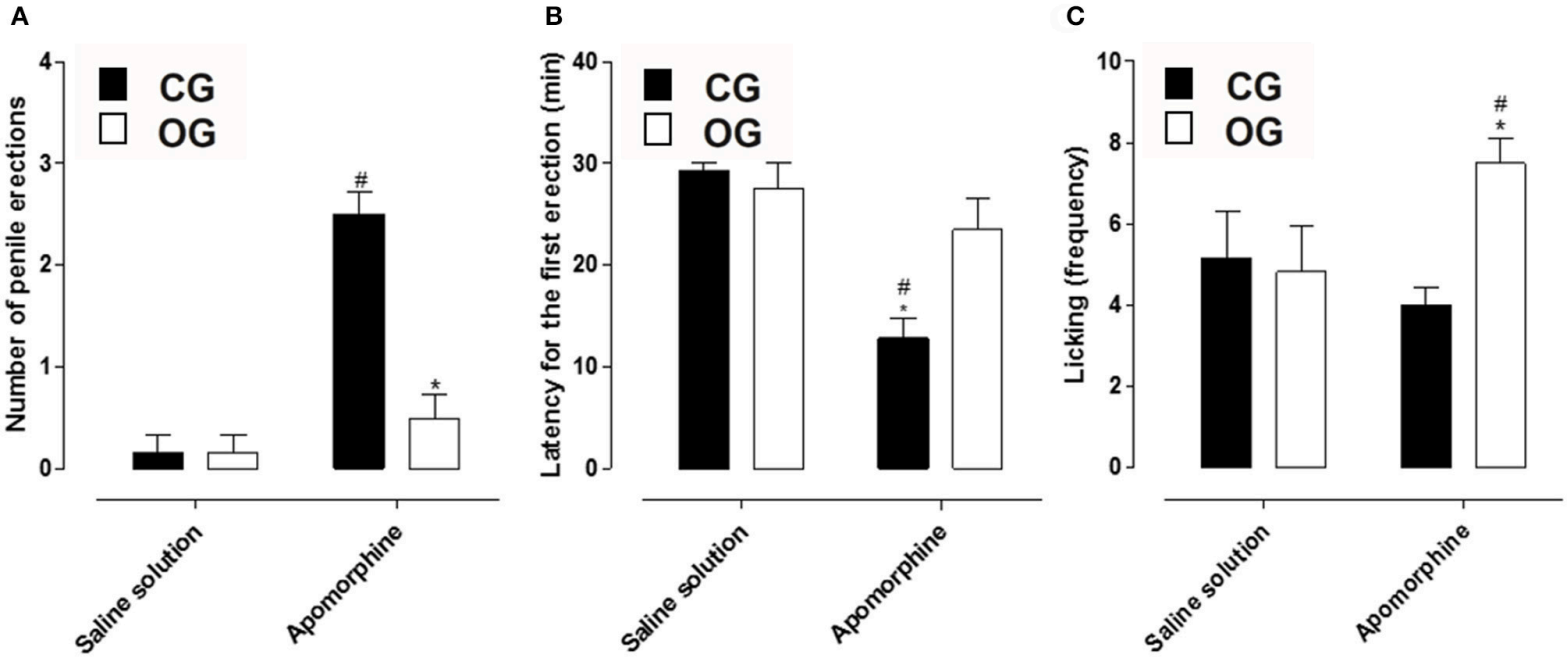
Number of penile erections **(A)**, latency for the first penile erection (min) **(B)** and licking events (frequency) **(C)** of rats from both CG (■) and OG (□). The columns and vertical bars represent the mean and S.E.M., respectively (*n* = 6). One-way ANOVA followed by Tukey's post-test, ^#^*p* < 0.05 (CG + saline solution vs. CG + apomorphine and OG + saline solution vs. OG + apomorphine) and ^*^p < 0.05 (CG apomorphine vs. OG + apomorphine).

Animals from CG that received apomorphine showed a penile erection's number of 2.5 ± 0.2, differing from those that received the saline solution (0.2 ± 0.2). However, animals from OG that received apomorphine presented a penile erection's number of 0.5 ± 0.2, and there was no difference between those that received the saline solution (0.2 ± 0.2). Based on the number of erections obtained in animals of both groups, a decrease in this parameter was verified in OG (Figure 5A, *n* = 5, one-way ANOVA followed by Tukey's post-test).

Additionally, the latency to start the first erection in animals from the CG, in the presence of apomorphine, was 12.8 ± 1.9 min, differing from those that received saline solution (29.2 ± 0.8 min). Similarly, the latency for the first erection of animals from the OG that received apomorphine was 23.5 ± 3.1 min, not differing from those receiving saline 27.0 ± 3.0 min. Withal, when comparing both groups, a decrease in the time required to start the first penile erection was verified in OG (Figure 5B, *n* = 5, one-way ANOVA followed by Tukey's post-test).

Besides that, the lick frequency of the CG in the presence of apomorphine (4.0 ± 0.4) was similar to the CG in the presence of saline solution (5.2 ± 1.4). Also, the licking frequency of OG in the presence of apomorphine (7.5 ± 0.6) presented difference when compared to the animals in this group that received saline solution (4.8 ± 1.3). Comparing both groups, in the presence of apomorphine, OG showed a higher number of licks compared to the other groups (Figure 5C, *n* = 5, one-way ANOVA followed by Tukey's post-test).

### Correlation between anthropometric parameters and the number of penile erections

**Figure 6:**
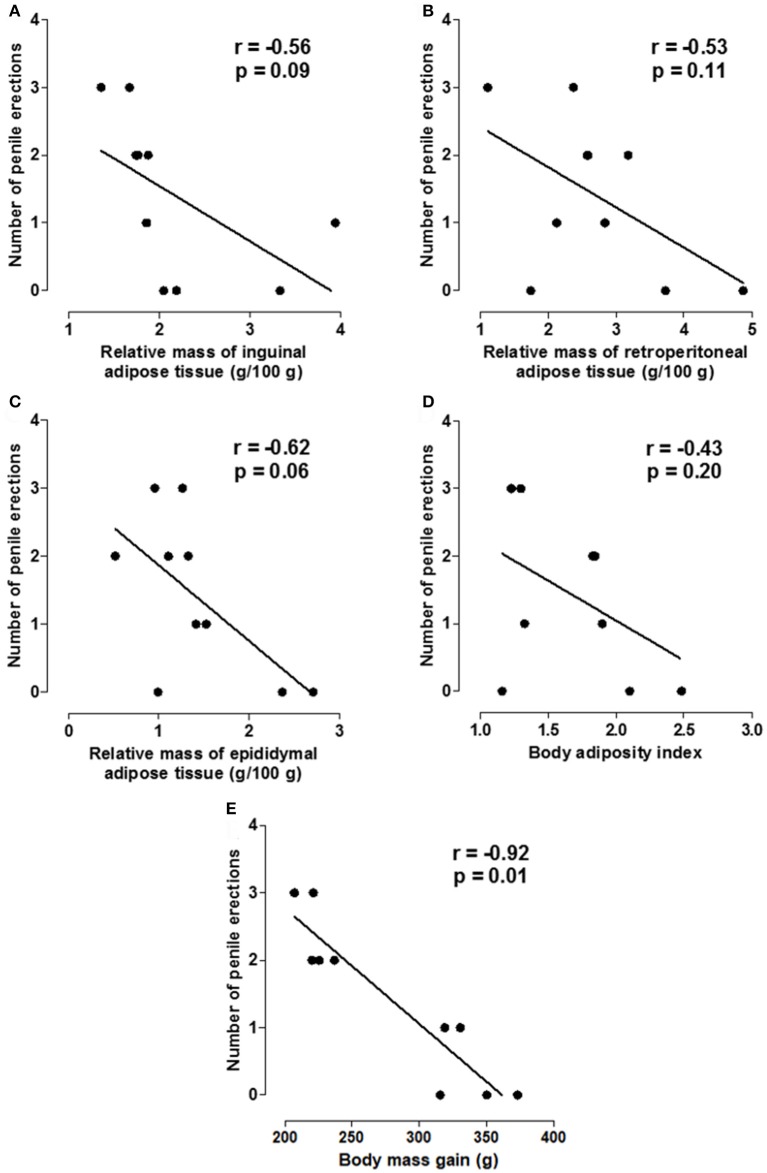
Correlation between the number of erections and the relative mass of inguinal **(A)**, retroperitoneal **(B)** and epididymal adipose tissues (g/100 g) **(C)**, body adiposity index **(D)** and body mass gain **(E)**.

The negative correlation of the number of penile erections with the relative mass of inguinal (*r* = −0.56, *p* = 0.09), retroperitoneal (*r* = −0.53, *p* = 0.11) and epididymal adipose tissues (*r* = −0.62, *p* = 0.06) as well as the body adiposity index (*r* = −0.43, *p* = 0.20) was not statistically significant. However, there was a strong negative correlation between the number of penile erections and body mass gain (*r* = −0.92, *p* = 0.01) (Figure 6, *n* = 10, Pearson's correlation).

### Contractile reactivity measurement

**Figure 7:**
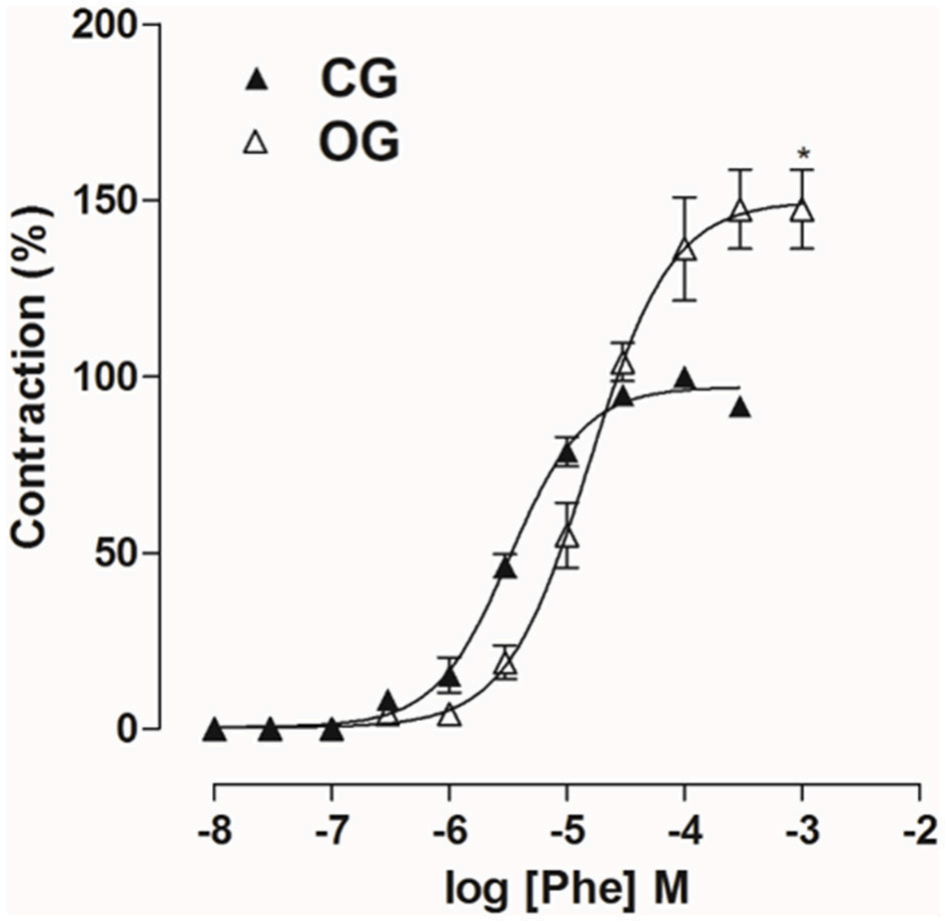
Cumulative concentration-response curves to Phe (10^−8^–10^−3^ M) on rat corpus cavernous from both CG (▴) and OG (▵). The symbols and vertical bars represent the mean and S.E.M., respectively (*n* = 5). Student's *t*-test, ^*^p < 0.05 (CG vs. OG).

In OG, cumulative concentration-response curves to Phe (10^−8^–10^−3^ M) were shifted to the right (pD_2_ = 4.8 ± 0.06), compared to CG (pD_2_ = 5.5 ± 0.06). In addition, the E_max_ obtained was increased in the OG compared to CG (E_max_ = 147.5 ± 11.2 and 100%, respectively). The concentration of Phe that produced its E_max_ in CG was 10^−4^ M, while in OG it was 3 × 10^−4^ M. In addition, the concentration that produced the submaximal effect (approximately 80%) was 10^−5^ M in CG and 3 × 10^−5^ M in OG (Figure 7, *n* = 5, Student's *t*-test).

**Figure 8:**
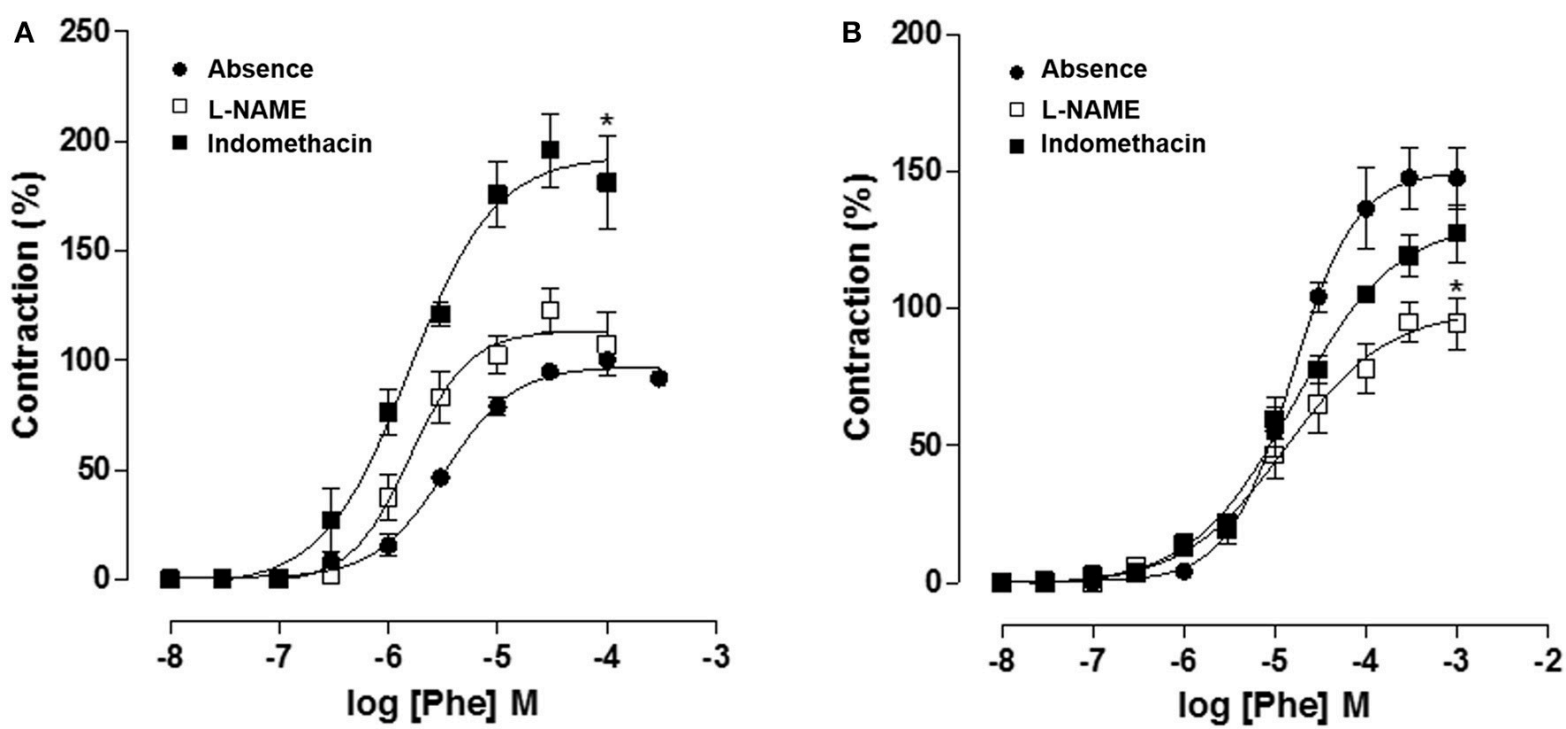
Cumulative concentration-response curves to Phe (10^−8^–10^−3^ M) in both absence (●) or presence of indomethacin 10^−5^ M (□) or L-NAME 10^−4^ M (■) on rat corpus cavernous from both CG **(A)** and OG **(B)**. The symbols and vertical bars represent the mean and S.E.M., respectively (*n* = 5). One-way ANOVA followed by Tukey's post-test, ^*^p < 0.05 (Phe vs. indomethacin + Phe and Phe vs. L-NAME + Phe).

Moreover, in CG, cumulative concentration-response curves to Phe (10^−8^–10^−3^ M) (E_max_ = 100%; pD_2_ = 5.5 ± 0.06) were shifted to the left in a non-parallel manner with increase in both E_max_ and pD_2_, in the presence of L-NAME 10^−4^ M (E_max_ = 195.9 ± 16.7%; pD_2_ = 5.8 ± 0.1). However, cumulative curves to Phe were not altered in the presence of indomethacin 10^−5^ M (E_max_ = 122.9 ± 10.1%; pD_2_ = 5.7 ± 0.07) (Figure 8A, *n* = 5). Meanwhile, in OG, cumulative curves to Phe (E_max_ = 147.5 ± 11.2%; pD_2_ = 4.8 ± 0.06) did not differ from that obtained in the presence of L-NAME 10^−4^ M (E_max_ = 119.3 ± 7.6%; pD_2_ = 4.9 ± 0.1). In addition, Phe efficacy was reduced in the presence of indomethacin 10^−5^ M (E_max_ = 94.9 ± 7.2%; pD_2_ = 4.9 ± 0.1) (Figure 8B, *n* = 5, one-way ANOVA followed by Tukey's post-test).

### Relaxing reactivity measurement

**Figure 9:**
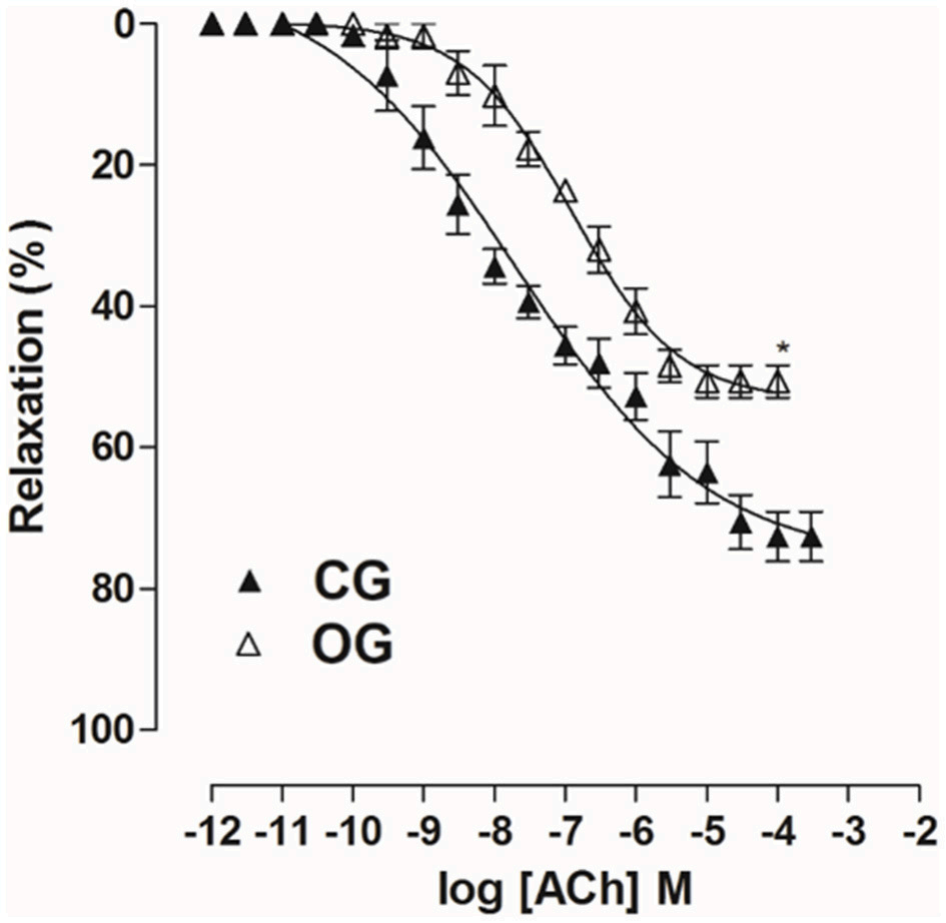
Cumulative concentration-response curves to ACh (10^−12^–10^−3^ M) on rat corpus cavernous pre-contracted with Phe 10^−5^ M from both CG (▴) and OG (▵). The symbols and vertical bars represent the mean and S.E.M., respectively (*n* = 5). Student's *t*-test, ^*^p < 0.05 (CG vs. OG).

**Figure 10:**
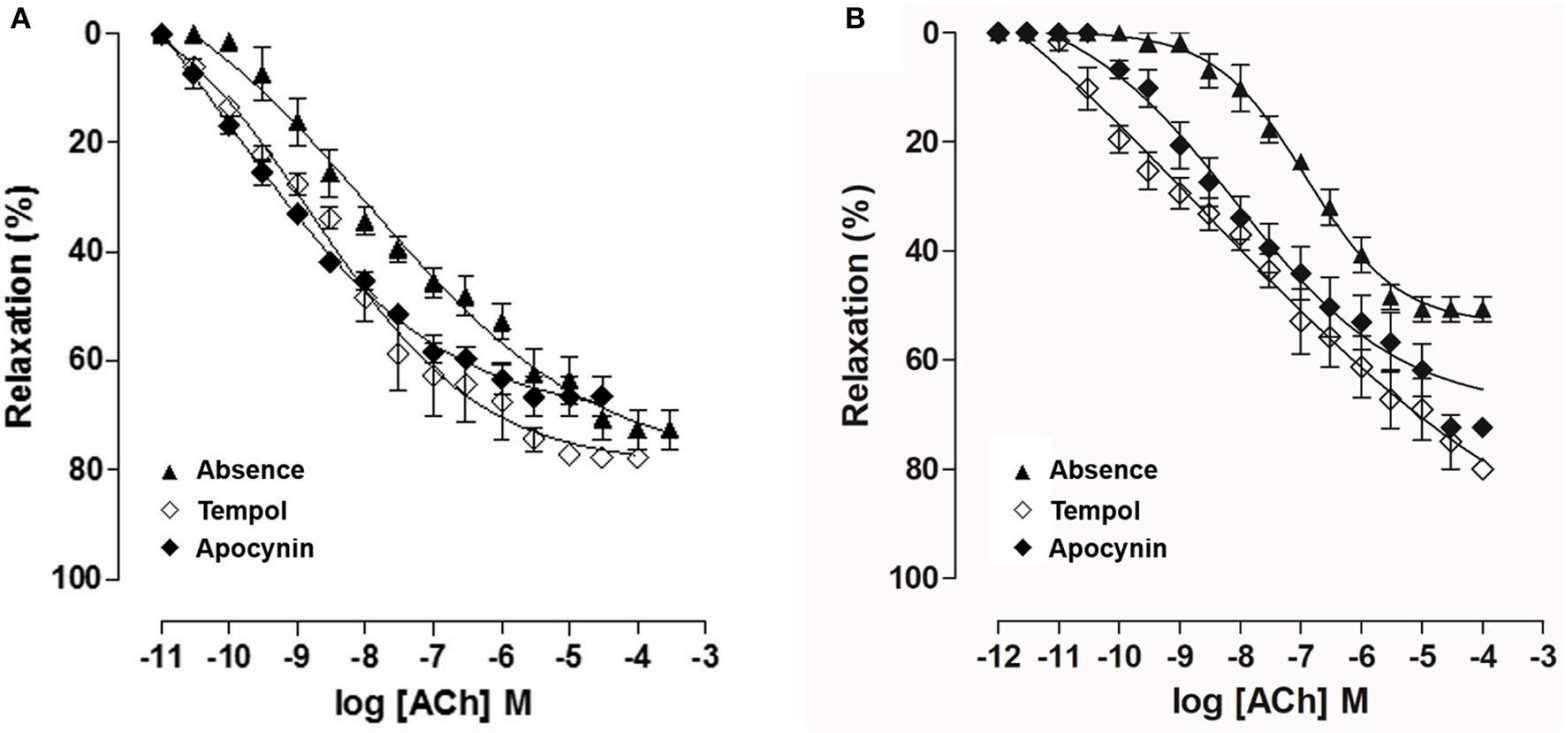
Cumulative concentration-response curves to ACh (10^−12^–10^−3^ M) in both absence (▴) or presence of tempol 10^−3^ M (♢) or apocynin 10^−4^ M (♦) on rat corpus cavernous from both CG **(A)** and OG **(B)**. The symbols and vertical bars represent the mean and S.E.M., respectively (*n* = 5). One-way ANOVA followed by Tukey's post-test.

In OG, cumulative concentration-response curves to ACh (10^−11^–10^−4^ M) were shifted to the right (pD_2_ = 7.0 ± 0.05), compared to CG (pD_2_ = 7.6 ± 0.1). Moreover, the E_max_ obtained was decreased in the OG compared to CG (E_max_ = 50.7 ± 2.3 and 72.6 ± 3.5%, respectively) (Figure 9, *n* = 5). However, in both CG and OG, ACh potency was 13 and 23-fold increased in the presence of tempol 10^−3^ M, (pD_2_ = 8.8 ± 0.1 and 8.5 ± 0.1, respectively), and both 19-fold increased in the presence of apocynin 10^−4^ M, (pD_2_ = 8.9 ± 0.1 and 8.3 ± 0.2, respectively) (Figure 10, *n* = 5, Student's *t*-test).

### Assessment of lipidic peroxidation levels

The MDA levels in rat plasma was increased from 5.3 ± 0.2 μM/L (CG) to 6.8 ± 0.4 μM/L in OG (*n* = 10, Student's *t*-test).

### Evaluation of antioxidant activity

The percentage of inhibition of plasma oxidation in rat plasma was decreased from 44.4 ± 1.2% (CG) to 34.0 ± 2.7% in OG (*n* = 10, Student's *t*-test).

## Discussion

Obesity is a serious public health problem and has long been considered as one of the most important nutritional disorders in developed countries (Dyer, 1994), with a growing incidence. Therefore, highlighting the interest in the study of biochemical and metabolic processes involved in the pathophysiology of obesity in order to provide an effective treatment (Naves and Paschoal, 2007). In addition, due to the ethical limitation in studying the susceptibility of humans to obesity, animal models of obesity have been proposed to investigate the alterations caused by this disease (Pereira et al., 2003).

Obesity has been induced in animals through neural damage, endocrine, genetic and/or eating disorders (Sclafani and Springer, 1976). In this study, obesity was induced in rats through dietary manipulation, a process that best reproduces the human obesity (Meguid et al., 2004). The experimental diet offered to OG presented a higher percentage of lipid and energy value than the diet consumed by CG (Table 1), being characterized as a hypercaloric diet, as observed in studies using a similar diet (Estadella et al., 2011).

In different experimental models, the period of a hypercaloric diet consumption is varied, most of them included in the range between 4 and 20 weeks, predominantly, the protocols used 8 weeks to rats (Rosini et al., 2012). Thus, in a 8-week period, it was observed that the OG had lower food consumption than the CG as well as a lower caloric intake (Tables 2–4). This fact is justified by the higher energy density of hypercaloric and hyperlipidic diets, which results in a greater satiety (Hariri and Thibault, 2010). Similar results were observed in studies using the diet proposed by Estadella et al. (2004) in both male and female rats (Bernardes et al., 2004). However, Eguchi et al. (2008) did not observe a change in animal's food consumption.

Besides the decrease in food and caloric intake was observed an increase in OG body mass. In order to guarantee that any difference in the body mass gain of the animals used in this study was associated to the consumption of differentiated diets, both OG and CG started the experiments with the same mass range. However, at the end of the experiment period, OG presented a greater body mass compared to CG (Figure 1) related to a higher energy density of diet offered to OG, corroborating other studies that used the same diet in both male and female rats (Nascimento et al., 2008; Ravagnani et al., 2012).

Despite the difference found in the CG and OG body mass, the characterization of the obesity development should address different parameters such as Lee index, BMI, relative mass of the adipose tissue deposits and the body adiposity index, with the purpose of providing information on the distribution of body fat (Yao et al., 2002).

The Lee index does not present a classificatory level, differing from the BMI that is applicable to humans, but the animals that presented a higher value of this index are more likely to develop obesity. There are studies that demonstrate a difference in Lee index in obese animals (Junqueira et al., 2011). However, in the present study the Lee index did not differ in the experimental groups, in a similar situation observed by Nery et al. (2011) and Malafaia et al. (2013).

Due to the inconsistent variation of the Lee index values in many obesity studies, the correlation between the BMI and the carcass lipid composition of rats was validated, demonstrating the importance of this index in the estimation of body fat and obesity, also in rats (Novelli et al., 2007). Meanwhile, in the present study BMI also did not differ in the experimental groups. The BMI of the animals ranged from 0.58 to 0.60 g/cm^2^, within the range considered normal for rats of 30–150 days of age (0.38–0.68 g/cm^2^) (Novelli et al., 2007). In fact, for some authors, the animal weight and length are unsatisfactory for estimating free adipose mass in animals of similar age (Stephens, 1980).

In humans, BMI is the main criterion used to confirm obesity; in addition, another aspect that stands out is the adiposity verified through the deposits of subcutaneous and visceral adipose tissues (Thibault et al., 2004). Thus, a number of methods used to determine obesity in animals were used to quantify the deposits of visceral (retroperitoneal and epididymal) and subcutaneous (inguinal) adipose tissues, as well as the body adiposity index (Woods et al., 2003). Based on this information, when comparing the relative (normalized) mass and the adipocyte diameters of the inguinal, retroperitoneal and epididymal adipose tissues, was observed an increase in animals from the OG compared to the CG (Figures 2–4), thus indicating an increase in central and visceral adiposity, as well as in the adiposity index, as verified in other studies with animal obesity (Schrauwen and Westerterp, 2000).

In view of the increase in the final body mass, adipose tissue deposits and the adiposity index of OG, the standardization of the obesity model was confirmed in these rats submitted to the consumption of the experimental hypercaloric diet for a period of 8 weeks. In addition, the diet consumed by the OG promoted obesity without changes in glucose, cholesterol and triglyceride levels.

Knowing that obesity is correlated with different diseases, among them ED, which affects millions of men worldwide and compromises their self-esteem and interpersonal relationship, negatively affecting their quality of life, the development of animal models have allowed significant advances in the field of this disease. However, the perfect model for study ED remains unclear, as the etiology of this disease is multifactorial. Mainly, animal's age, type and/or strain affect the outcome of the study of male sexual behavior (Chung et al., 2011).

It is well documented that human ED can be caused by aging, psychogenic or organic factors (involving vascular endocrine and neurological disorders) and even due to the use of medications (anxiolytics and antidepressants, for example) (Wagner and Mulhall, 2001). As a consequence of the increased correlation between obesity and erectile dysfunction in men, in this study was evaluated the effect of the consumption of the hypercaloric diet on the erectile function of Wistar rats. Data shown that between the 8th and 10th week of life rats reach sexual maturity, and are considered young adults (Baker et al., 1979), thus, it was decided to start our experiments using animals at 8 weeks of age.

The use of rat for ED studies has increased, due to the similarity to human's penis in morphological, functional, vascularization and peripheral neural supply. In fact, there is a high transposition from rat ED model to human disease. Meanwhile, the human sexual response involves more visual and auditory stimulation; the rodents have a major olfactory stimulation. Therefore, the complexity of human sexual behavior and ED cannot be fully represented by a specific animal's model of ED (Chung et al., 2011).

To avoid the limitation of different stimuli that evoke erection in rats, apomorphine was used as a penile erection inducing agent. It is a non-selective dopaminergic agonist, which shows greater selectivity for the D_2_-like receptors present in the paraventricular nucleus (PVN), where it activates a cascade of intracellular biochemical events, activating the NOS enzyme and, as consequence, an increase in NO synthesis, which stimulates the release of oxytocin in other regions of the central and peripheral nervous system (Matsumoto et al., 2005). In this signaling pathway, the spinal cord is highlighted, which contains oxytocinergic fibers that originate in PVN. In addition, the parasympathetic neurons in the lumbosacral region innervated the corpus cavernosum and they respond to an oxytocinergic stimulation. Therefore, the oxytocin released during PVN activation is a potent activator of pro-erectile spinal neurons, leading to the cholinergic fibers activation on S2-S4 regions, and release of ACh in the corpus cavernosum, promoting penile erection (Marson and Mckenna, 1996; Giuliano and Rampin, 2000; Giuliano et al., 2001).

Using a methodology that evaluates erectile function in conscious animals, the rats received apomorphine and were recorded for a period of 30 min, as this drug is rapidly absorbed and transported through brain tissue. In addition, peak brain levels were reached within 15 min after its administration and subsequently its plasma concentration is reduced by half in 20 min (Melzacka et al., 1979; Urba-Holmgren et al., 1982; Hsieh et al., 2004; Hannan et al., 2006). The OG that received apomorphine did not present a difference in the number of penile erections compared to OG that received saline solution, but when comparing this group with the CG that received apomorphine, there was a decrease in the number of penile erections (Figure 5A), suggesting that the erectile function of animals submitted to a hypercaloric diet is impaired.

In addition, the time for the animals of both groups to present the 1st penile erection was different, CG time was about 15 min (Figure 5B), when the plasma concentration of apomorphine is maximal, corroborating the studies mentioned above. However, OG present a delay in the time to attempt to the 1^a^ penile erection (Figure 5B), corroborating that the erectile function of animals submitted to a hypercaloric diet is impaired. Another parameter analyzed was the frequency with which the animals licked the paws and abdomen, a common behavior in rats, however, increased and characteristic of the central effect of apomorphine (Carrier et al., 1997). All groups analyzed showed licking events, but there was an increase in the animals of OG (Figure 5C), demonstrating the efficacy of apomorphine in these animals due to the appearance of stereotyped behaviors such as licking and yawning (Rampin et al., 2003; Hannan et al., 2006).

Based on the analysis of the number of penile erections, latency for the first erection and licking frequency, it is possible to propose the standardization of a model of ED caused by the exposure of the animals to a hypercaloric diet, corroborating the studies that associate ED to obesity development (Alves et al., 2012; Aboua et al., 2014). Moreover, it was demonstrated a correlation between the increase in body mass gain and a decrease in the number of penile erections (Figure 6).

Once the erectile function of these animals was damaged *in vivo*, it was hypothesized that the contractile and relaxing reactivity of the corpus cavernous (*in vitro*) could be altered because it is well established in the literature that in obese animals there is an endothelial damage (Rosini et al., 2012), thus, it was performed contraction and relaxation curves in the corpus cavernosum of animals submitted to the standard and hypercaloric diets.

The relative contractile potency of Phe was attenuated in OG compared to CG. However, the maximal contractile response was increased in the OG (Figure 7). An increase in Phe efficacy was also observed in corpus cavernosum of obese mice (Toque et al., 2010). Based on this, we can correlate these results with the decrease in the number of penile erections in OG (Figure 5A), since the Phe efficacy increased may favor the maintenance of the penis in its flaccid state in these animals.

**Figure 11:**
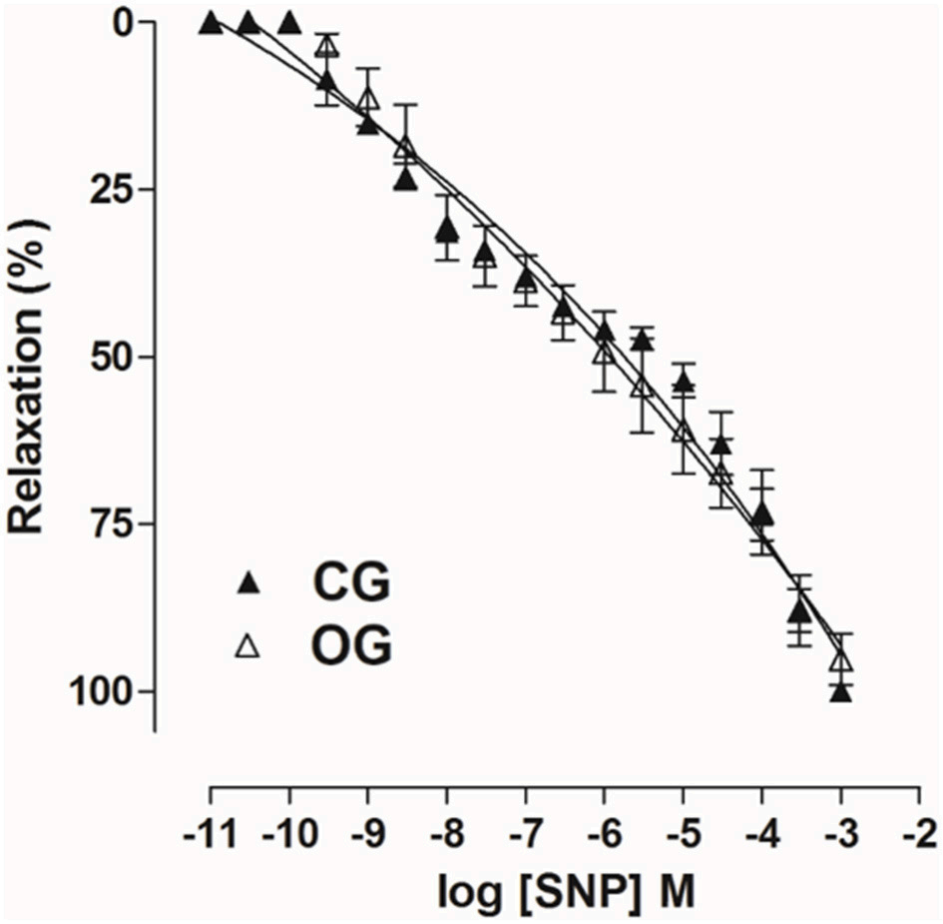
Cumulative concentration-response curves to SNP (10^−11^–10^−3^ M) on rat corpus cavernous pre-contracted with Phe 10^−5^ M from both CG (▴) and OG (▵). The symbols and vertical bars represent the mean and S.E.M., respectively (*n* = 5). Student's *t*-test.

Furthermore, cumulative concentration-response curves to SNP obtained in CG (pD_2_ = 5.9 ± 0.05 and E_max_ = 100%) were not altered in OC group (pD2 = 6.1 ± 0.2 and E_max_ = 96.7 ± 2.9%) (Figure 11, *n* = 5, one-way ANOVA followed by Tukey's post-test).

In addition, using L-NAME, was verified an increase in Phe values of E_max_ and pD_2_ in CG, as expected, the presence of NO impairs corpus cavernosum contraction. However, no changes were registered in the presence of indomethacin, suggesting no involvement of prostanoids on penile detumescence (Figure 8A). Meanwhile, in OG, Phe curves did not differ from that obtained in the presence of L-NAME, suggesting an endothelial damage due to reduce in NO bioavailability. Additionally, Phe efficacy was reduced in the presence of indomethacin, indicating an increase in contractile prostaglandins levels as a consequence of endothelial damage (Figure 8B).

Then, the alterations in the relaxing response of the rat corpus cavernosum was evaluated, for that, it was used ACh and SNP as pharmacological tools, with the purpose of identifying changes in the endothelial and muscular components, respectively. The OG showed a decrease in the relaxation promoted by ACh compared to CG (Figure 9), thus, indicating a possible endothelial damage in this organ, corroborating the result obtained in the *in vivo* erectile function experiments (Figure 5). The occurrence of endothelial dysfunction has also been reported in the corpus cavernosum of obese mice (Toque et al., 2010).

Comparing the displacement ratio of the relaxation curves of ACh in the presence of tempol in both groups, ACh potency was 13-fold increased in CG and 23-fold increased in OG (Figure 10A), highlighting the involvement of the superoxide anion (O_2_^•^) in the impairment of corpus cavernosum relaxation in obese rats. Therefore, O_2_^•^ seems to be involved in endothelial damage development in rats fed with a hypercaloric diet. However, the displacement ratio of the relaxation curves of ACh was similar in the presence of apocynin (Figure 10B). Thus, we can conclude that besides NADPH oxidase, other sources seem to be involved in the O_2_^•^ in obese rat, such as xanthine oxidase and eNOS uncoupling (Aitken et al., 1993; Yang et al., 2009).

However, by inducing an endothelium-independent relaxation with SNP, an NO donor, the relaxant response was not altered in OG relative to CG (Figure 11), indicating that there was possibly no damage to smooth muscle cells, and that ED observed in this study is not associated to muscle damage, contrary to that observed in models of neurogenic and arteriogenic ED (Lee et al., 2002; User et al., 2003; Ferrini et al., 2009).

Obesity development can lead to an increased production of reactive oxygen (ROS) and nitrogen (NRS) species, causing oxidative stress, and contributing for the NO bioavailability decrease (Esposito et al., 2004). Thus, it was observed that the hypercaloric diet consumption increase the oxidative stress, as can be seen by the increase in MDA production in OG compared to CG and the decrease in the percentage of inhibition of plasma oxidation, suggesting a possible change in the lipid matrix and cell membranes due to the effect of ROS production as well as the reduction of NO bioavailability (Chong et al., 2014).

Therefore, while the limitation of direct translation of rat ED to human condition, in the current study was established the development of ED in rats triggered by a low cost hypercaloric diet consumption and characterized by endothelial dysfunction associated to a decreased NO bioavailability, increased contractile prostaglandins production and O_2_^•^ presence. Thus, providing a model for advances in sexual dysfunction field and drug discovery for ED treatment.

## Author contributions

IdS performed literature search and pharmacological experiments, analyzed the data and wrote the paper; BB and GdO were involved in acquisition of functional pharmacological experiments; FQ and PS were involved in acquisition, interpretation and analysis of histological experiments; LT and AS were involved in acquisition, interpretation and analysis of biochemical and oxidative stress profile experiments; LI was involved in production and analysis of the hyperlipidic diet and interpretation of nutritional data; FC and BdS were involved in design, interpretation of the data and paper review.

### Conflict of interest statement

The authors declare that the research was conducted in the absence of any commercial or financial relationships that could be construed as a potential conflict of interest.
